# Genome-wide positioning of bivalent mononucleosomes

**DOI:** 10.1186/s12920-016-0221-6

**Published:** 2016-09-15

**Authors:** Subhojit Sen, Kirsten F. Block, Alice Pasini, Stephen B. Baylin, Hariharan Easwaran

**Affiliations:** 1 CRB1, Room 530, Department of Oncology and The Sidney Kimmel Comprehensive Cancer Center at Johns Hopkins, The Johns Hopkins University School of Medicine, Baltimore, 21287 MD USA; 2 UM-DAE Centre for Excellence in Basic Sciences, University of Mumbai, Kalina Campus, Santacruz (East), Mumbai, 400098 India; 3 Division of Respiratory Medicine and Nottingham Respiratory Biomedical Research Unit, University of Nottingham, City Hospital, Nottingham, NG5 1BP UK

**Keywords:** Bivalent mononucleosomes, Bivalency, DNA methylation, Chromatin, H3K4me3, H3K27me3

## Abstract

**Background:**

Bivalent chromatin refers to overlapping regions containing activating histone H3 Lys4 trimethylation (H3K4me3) and inactivating H3K27me3 marks. Existence of such bivalent marks on the same nucleosome has only recently been suggested. Previous genome-wide efforts to characterize bivalent chromatin have focused primarily on individual marks to define overlapping zones of bivalency rather than mapping positions of truly bivalent mononucleosomes.

**Results:**

Here, we developed an efficacious sequential ChIP technique for examining global positioning of individual bivalent nucleosomes. Using next generation sequencing approaches we show that although individual H3K4me3 and H3K27me3 marks overlap in broad zones, bivalent nucleosomes are focally enriched in the vicinity of the transcription start site (TSS). These seem to occupy the H2A.Z nucleosome positions previously described as salt-labile nucleosomes, and are correlated with low gene expression. Although the enrichment profiles of bivalent nucleosomes show a clear dependency on CpG island content, they demonstrate a stark anti-correlation with methylation status.

**Conclusions:**

We show that regional overlap of H3K4me3 and H3K27me3 chromatin tend to be upstream to the TSS, while bivalent nucleosomes with both marks are mainly promoter proximal near the TSS of CpG island-containing genes with poised/low expression. We discuss the implications of the focal enrichment of bivalent nucleosomes around the TSS on the poised chromatin state of promoters in stem cells.

**Electronic supplementary material:**

The online version of this article (doi:10.1186/s12920-016-0221-6) contains supplementary material, which is available to authorized users.

## Background

Different states of gene expression, ranging from silenced to fully expressed, are tightly controlled by chromatin structure and a concert of epigenetic regulators which act upon chromatin. Local epigenetic control lies in positioning of nucleosomes around transcription start sites (TSS) and control regions, along with post-translational modifications of histone tails and/or the presence of non-canonical histone variants such as H2A.Z and H3.3 [1, 2]. These dynamics exert a combinatorial effect on gene expression states alone or in sync with differential states of DNA methylation at CpGs throughout the genome [3–6]. Associated with the promoters of ~2500 genes in embryonic stem cells (ESC), ‘bivalent chromatin’ is defined by simultaneous presence of two opposing chromatin modifications, activating trimethylation at histone 3 lysine 4 (H3K4me3) and silencing trimethylation of histone 3 lysine 27 (H3K27me3) [7–9]. Bivalent chromatin correlates with a transcriptionally poised state which allows for plasticity in gene expression. Upon differentiation, genes with such chromatin often resolve to a monovalent state. In cancer, subsets of these genes identified as polycomb/bivalent marked in stem cells are often epigenetically silenced by cancer-specific *de novo* gains of promoter DNA methylation [10–13]. Therefore, it is important to understand the precise constitution of the chromatin states with respect to normal and abnormal gene expression, especially bivalent chromatin.

Initial characterization of bivalent chromatin described a “zonal” phenomenon in which a broad domain of H3K27me3 surrounds a more narrow occupancy of the H3K4me3 mark near the TSS [14]. What has since been unclear is whether, within this zonal context, the two marks reside simultaneously on the same nucleosome. A recent paper suggests that both the marks can co-exist on the same nucleosome but on different H3 peptides [15]. However, how many nucleosomes of this nature exist relative to zonal bivalency, and very importantly, what genomic positions these occupy, remains unknown.

In the present study, using a modified technique for sequential ChIP, we elucidate the genomic positioning of individual bivalent nucleosomes and their relations to other aspects of epigenetic regulation. We report a marked bias of bivalent nucleosomes immediately flanking the TSS exclusively in genes with proximal promoter CpG islands which are unmethylated. Intriguingly, we observe a relationship between these individual bivalent nucleosomes and previously established salt-labile nucleosomes at transcription start sites known to contain the variant histones H2A.Z and H3.3, which are key to initiation of transcription [16].

## Results

### Mononucleosome purification

A critical aspect of our study is the requirement of highly pure mononucleosomes in large quantities, enough for genome-wide analysis. Traditionally, most maps of histone modifications are created by performing ChIP on formaldehyde-crosslinked chromatin fractionated either by sonication or limited digest with micrococcal nuclease (MNase). While valuable, such maps have reduced resolution, owing to the generation of mixed populations of multinucleosomal fragments, since each modification could be pulled down sequentially on a substrate where these marks reside on neighboring nucleosomes. Therefore the only way to distinguish truly bivalent nucleosomes from heterogeneity within a cell population is to use sequential ChIP analyses of the two different marks on mononucleosome substrates. Hence, we aimed to obtain highly purified mononucleosomes as substrate and modified the sequential ChIP protocol for use in massively parallel sequencing.

**Fig. 1:**
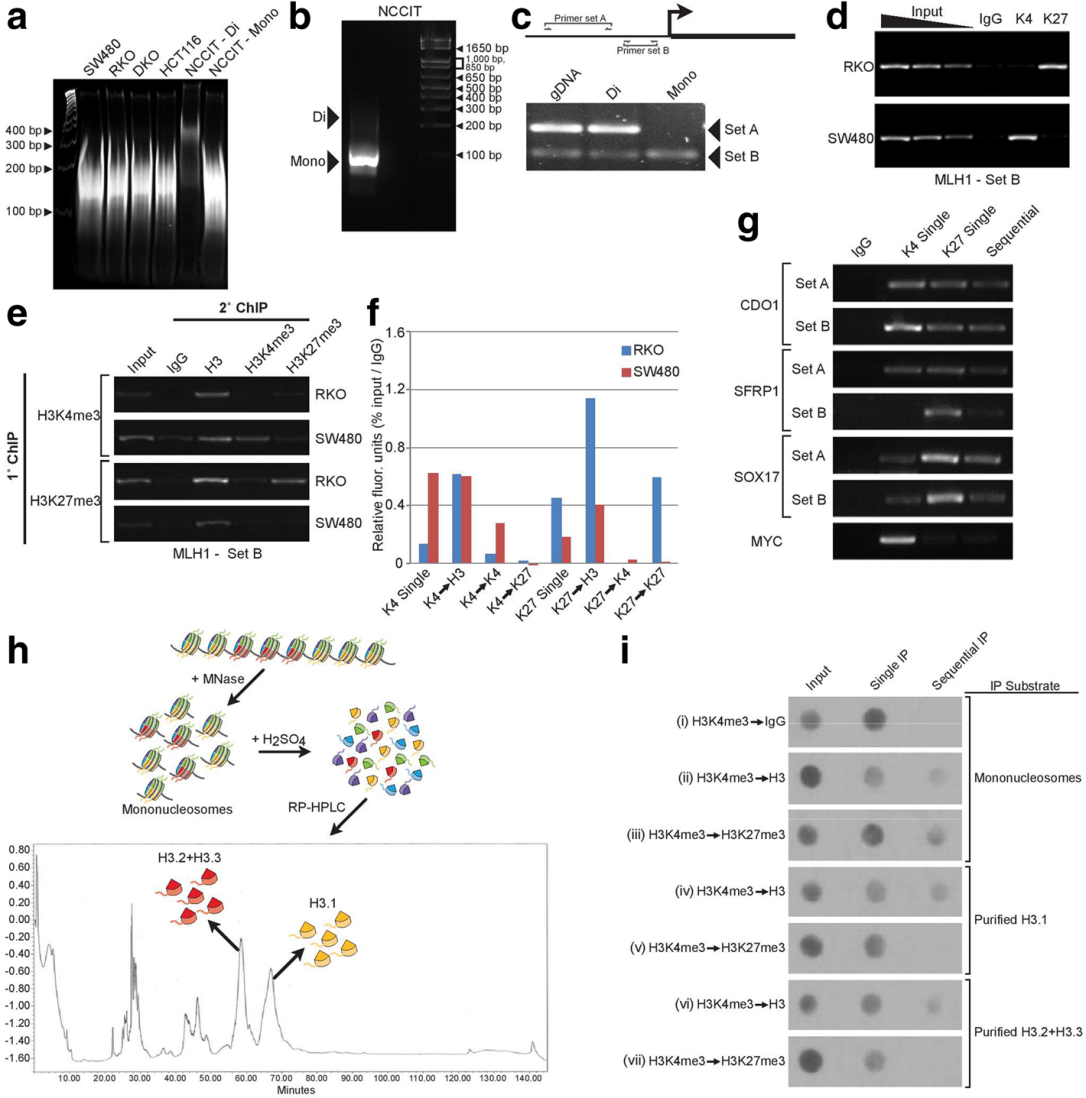
Optimization of mononucleosome isolation and sequential ChIP. **a** Mononucleosomes were isolated from the indicated cell lines by sucrose gradient (S.G.), and assessed for purity by native PAGE analysis. Dinucleosomal fragments from NCCIT (NCCIT-Di) were included for size reference. **b** A quick protocol (Q.P.) was developed (see Experimental Procedures), and resulting mononucleosomes from NCCIT cells were assessed for purity by overloading of DNA onto an agarose gel. **c** Top shows a schematic representation of the TSS region of MLH1 with placement of primer pairs. Below, PCR analysis of mononucleosomes (mono, from Q.P.), dinucleosomes (di, from S.G.) and genomic DNA (gDNA) of NCCIT cells. **d** Mononucleosomes isolated from RKO and SW480 (using Q.P.) were subjected to ChIP, MLH1 promoter analysis (primer set described above, part C). **e** Mononucleosomes isolated from RKO and SW480 (using Q.P.) were subjected to sequential ChIP using indicated combinations of anti- H3, H3K4me3 and H3K27me3 immunoprecipitation (IP), and the MLH1 promoter was analyzed by primer set-B described above. **f** Amplicons depicted in E was quantitated by ImageJ (all data expressed as percentage of input and normalized to IgG background). **g** Mononucleosomes from NCCIT cells (using Q.P.) were subjected to single or sequential ChIP (precipitation of H3K27me3 followed by H3K4me3). Chromatin patterns of *CDO1*, *SFRP1* and *SOX17* were assessed between treatment groups and compared to *MYC* as a control. **h** The schematic depicts isolation of mononucleosomes and individual histone peptides, the latter as depicted by peaks in the HPLC analysis below. **i** Sequential IP of mononucleosomes (using Q.P.) or purified histone peptide substrates from NCCIT cells were analyzed by dot blot using anti-H3K4me3 antibody (left panel shows sequence of IP)

Traditional purification protocols extract nucleosomes under physiological salt concentrations (100–150 mM NaCl). However, nucleosomes closer to the transcription start sites, containing histone variants H3.3 and H2A.Z are labile at these salt conditions [17]. The mononucleosome preparation protocols used herein aimed to minimize the potential loss of these salt-labile nucleosomes by low salt extraction (20 mM NaCl, [17]) and immediately crosslinked them at low concentrations of formaldehyde to fix nucleosome positions whilst preventing inter-crosslinking of mononucleosomes and non-specific aggregates (data not shown). Mononucleosomes were then purified to homogeneity using a 5–25 % sucrose density gradient (Additional file 1: Figure S1) and further analyzed for purity and lack of dinucleosomes. Using this approach, we were able to detect only mononucleosomal bands, and in spite of gel overloading, no dinucleosomal DNA was observed (Fig. 1a).

Because large amounts of mononucleosomes were needed for sequential ChIP, an alternative “quick protocol” was also developed for mononucleosome isolation, the purity of which was similar to the rigorous controls above (Fig. 1b). The quick protocol relies upon differential elution of mononucleosomes from nuclei with restrictive detergent concentrations of TritonX-100 and IGEPAL ca630 followed by immediate crosslinking by formaldehyde. The absence of contaminating dinucleosomal DNA in these preparations was determined both by gel analysis (Fig. 1b) and PCR at *MLH1* promoter, which revealed that amplicons larger than 147 bp were undetectable (Fig. 1c).

### Existence of bivalency at the mononucleosome level but not on same H3-peptide tail

To characterize the efficacy of sequential ChIP, first single ChIP assays were performed and compared with a series of different combinations of sequential ChIPs for stringent controls, using crosslinked mononucleosomes as substrate. The efficacy was tested using the duality of histone modifications at *MLH1* gene promoter in human colorectal cancer (CRC) cell lines RKO (silenced, H3k27me3 enriched) versus SW480 (expressed, H3K4me3 enriched), as established from previous work [18]. *MLH1* is silenced in RKO and associated with both abnormal CpG island promoter DNA methylation as well as some H3K27me3 enrichment. Contrasting this, *MLH1* is highly active and marked by H3K4me3 in SW480. Indeed, we observed identical results when both sucrose gradient preparations and mononucleosomes obtained by quick-prep were probed using single ChIPs (Fig. 1d, results shown for ‘quick prep’).

Next we utilized these mononucleosome preparations for sequential ChIP (seq-ChIP) analyses in RKO and SW480, using the same *MLH1* promoter as reference. An important modification of the existing sequential ChIP protocol was that the primary ChIP (1°) antibody was crosslinked to protein A/G beads using disuccinimidyl glutarate (DSG), preventing its elution into the secondary ChIP (2°) step, thus increasing sensitivity and avoiding any ambiguity of analysis (Additional file 2: Figure S2A-B). Employing key controls, we find that primary IP (1°) against H3 followed by secondary IP (2°) using the same antibody (1°H3 → 2°H3, positive control) resulted in amplification of *MLH1* promoter DNA from SW480-derived mononucleosomes, while the combination of H3 pull-down followed by secondary IP with IgG (1°H3 → 2°IgG, negative control) in the same population yielded no enrichment (Additional file 2: Figure S2C). When we examined the transcriptionally active *MLH1* promoter in SW480, we observed enrichment only in sequential ChIP directed against 1°H3K4me3 → 2°H3 (or 1°H3K4me3 → 2°H3K4me3), used as positive controls, but no enrichment in samples subjected to 1° anti-H3K4me3 pull-down followed by 2° anti-H3K27me3 pull-down (Fig. 1e-f). An exact converse was revealed at the silenced *MLH1* promoter region in RKO cells which is enriched only when antibodies against 1°H3K27me3 → 2°H3 (or 1°H3K27me3 → 2°H3K27me3) are used, revealing a lack of H3K4me3 at the region analyzed. Thus, concluding from the test combinations of 1°H3K4me3 → 2°H3K27me3 antibodies or the reverse (1°H3K27me3 → 2°H3K4me3) pull down in either cell line demonstrated complete lack of enrichment of both marks, indicating that the *MLH1* promoter region examined lacks bivalent chromatin in both SW480 and RKO (Fig. 1f, fourth and seventh set in graph). Taken together, these analyses confirm that the assay developed is not only independent of the direction of antibody used for 1°ChIP but also specific in detecting only the monovalent chromatin configurations for the examined nucleosome in the *MLH1* promoter in the cells examined, negating any carryover effect of the 1° ChIP into the 2° sequential ChIP, a very important factor in discerning true bivalency.

Having confirmed seq-ChIP with stringent controls, we then embarked on detection of possible bivalent nucleosomes in the embryonic carcinoma line NCCIT as embryonic cell lines are undifferentiated and maintain multiple genes in the bivalent state. We used multiple primer sets designed to examine a panel of genes previously shown to carry bivalent chromatin in ES [9] and NCCIT cells [19]. When assayed for bivalent marks individually, we not only find promoter regions of *CDO1, SFRP1* and *SOX17*, genes (also known to be frequently DNA hypermethylated in cancer cells [20–22]), to be enriched for both H3K27me3 and H3K4me3 but also to display these marks concomitantly at the same mononucleosome when assayed for by sequential pull-down using 1°H3K27me3 → 2°H3K4me3 (Fig. 1g). Notably, the mononucleosome assessed by *SFRP1* primer set B showed enrichment of only H3K27me3, while primer set A indicated the presence of a bivalent nucleosome at that position in the same gene. This suggests probable heterogeneity for truly bivalent nucleosomes within the promoter region, which would otherwise be identified as a bivalent “domain”. In addition, as a control, the highly active *MYC* promoter, for the region examined, is enriched for only H3K4me3, showing no detectable amplicons in sequential ChIP following initial pull-down of H3K27me3. Thus in summary, our examination of genes in NCCIT, SW480 and RKO cells, indicate that the sequential ChIP protocol is indeed both sensitive and specific for bivalent mononucleosomes. These results also support previous proteomic discoveries of another group for the existence of truly bivalent mononucleosomes [15].

To help fortify the above results, we used an additional proteomics approach to examine the pull-down products from sequential ChIP to test if both marks co-exist on the same histone peptide. We purified the core histone H3.1 and its transcriptional variant H3.3 by HPLC [23] and then used these purified histone pools as substrates for sequential IP (Fig. 1h and i). In this peptide context, only those histones that carry both H3K27me3 and H3K4me3 on the same peptide tail would be isolated by a sequential IP. Purified refolded H3.1 and H3.3 histone substrates (panel’s iv–vii) as well as intact mononucleosomes substrates as controls (panel’s i–iii) were sequentially pulled down, and the eluted samples were analyzed for the presence of H3K4me3 using dot blots (Fig. 1i). For both purified H3.1 and H3.2/H3.3, sequential ChIP using 1°H3K4me3 → 2°H3 (Fig. 1i, panels iv and vi) yielded H3K4me3-marked peptides as expected. However, H3K4me3-marked peptides (Fig. 1i, Single IP column) subjected to secondary ChIP with antibodies directed against H3K27me3 were no longer observed in the final elution (Fig. 1i panels v and vii). These results indicate that, within the limits of detection, both marks may not co-exist on the same peptide. Taken together with the positive signal for sequential-IP with mononucleosome substrates under the same conditions (Fig. 1i panel iii), our results strongly support the model that both the marks exist asymmetrically on different histone peptides within the same mononucleosome context, as established before [15].

### Genome-wide position of bivalent nucleosomes versus individual H3K4me3 and H3K27me3 marks

We used ‘quick-prep’ purified mononucleosomes from NCCIT cells as a substrate for sequential-ChIP, in which nucleosomes isolated by anti-H3K27me3 pull-down were subsequently subjected to secondary ChIP by anti-H3K4me3, followed by massively parallel sequencing to examine genome-wide patterns of H3K4me3, H3K27me3, and bivalency at nucleosome resolution. In addition, we mapped H2A.Z as it is associated with the open chromatin region of active genes [16]. We performed the seq-ChIP using H3K27me3 as the primary antibody for two reasons; (i) to avoid any carryover of the dominant H3K4me3 mark (due to better antibody reactivity) into the sequential 2^o^ IP and (ii) several batches of anti-H3K4me3 antibodies were sensitive to crosslinking by DSG (unpublished results) introducing ambiguity in pull down activity, and hence can only be used in the 2°IP step (which doesn’t involve crosslinking). This restricted the directionality of seq-ChIP for scale up to genome wide platforms.

**Fig. 2:**
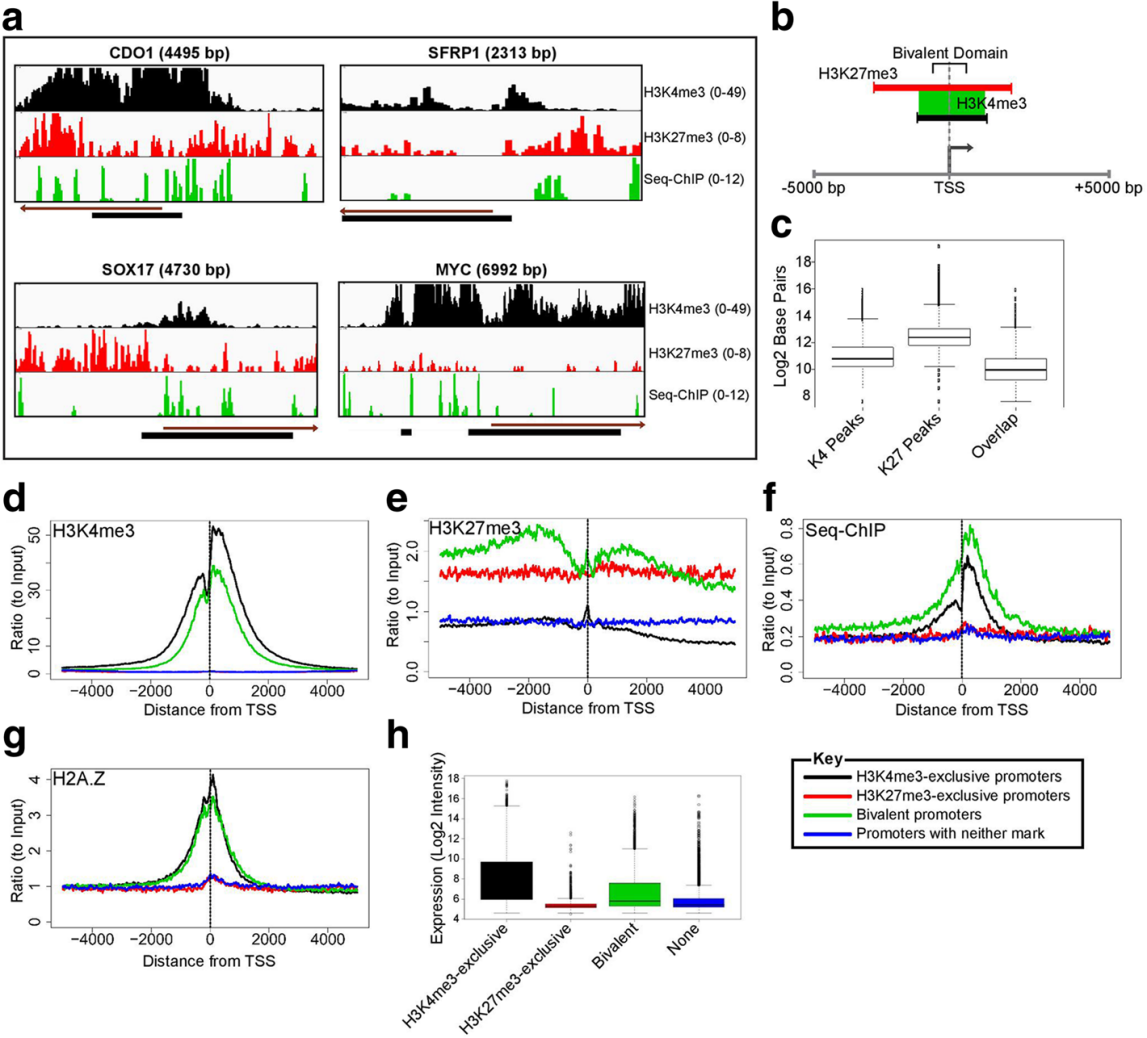
Distribution of bivalent nucleosomes at gene promoters. **a** Examples of individual genes showing the patterns of H3K4me3, H3K27me3 and bivalent nucleosomes around the TSS. Direction of gene transcription (*brown arrow*) and CpG islands (*black bar*) shown. **b** Schematic of distribution of H3K4me3 (*black*) and H3K27me3 (*red*) peaks called within +/−5000 bp from the TSS. Genes previously described as bivalent carry overlapping H3K4me3 and H3K27me3 (*green region*) peaks. **c** Size distribution of the H3K4me3, H3K27me3 peak calls, and the overlapping regions of these peaks. **d**–**g** Distribution of H3K4me3 **d** H3K27me3 **e** Sequential ChIP (H3K27me3 → H3K4me3)-seq reads (bivalent nucleosomes, **f**), and H2A.Z **g** around the TSS (x-axis, TSS is 0). Promoters were classified into H3K4me3-exclusive (*black*), H3K27me3-exclusive (*red*), or none (none of the marks detected), promoters based on identification of ChIP-seq enrichment peak calls using SICER in the +/−2500 bp around the TSS. Promoters with overlapping H3K4me3 and H3K27me3 enrichment peak calls in the +/−2500 bp around the TSS were defined as Bivalent promoters (*green*). ChIP-seq reads were binned at 10 bp intervals. Y-axis represents average ChIP-seq reads normalized to corresponding average of the input .**h** Gene expression ranges of the three different promoter classes

To provide initial context for our genome-wide analyses, we first viewed a series of candidate genes, including those analyzed by our local ChIP studies (Fig. 1g), for the genomic positions and amplitudes of the individual H3K4me3 and H3K27me3 marks that make up bivalency (Fig. 2a). Multiple studies have reported that H3K4me3 enrichment immediately flanks the TSS while H3K27me3 enrichment typically spans a broader region around the TSS [24, 25], which we verify for multiple genes (Fig. 2a and Additional file 3: Figure S3A–D). Both marks are enriched around the TSS, with H3K4me3 being markedly contiguous in the immediate vicinity of the TSS. Strikingly, despite overlapping signals in H3K4me3 and H3K27me3 ChIP-seq profiles for the selected genes, individual bivalent nucleosomes mapped by sequential ChIP-seq appear scattered around the TSS without a discernible pattern (Fig. 2a). This punctate occupancy of bivalent nucleosomes is in agreement with the earlier observation by PCR that not all nucleosomes within a classically defined “bivalent” zone are, in fact, bivalent (see *SFRP1* in Fig. 1g above).

To elucidate the general distribution of bivalent nucleosomes around TSS, we classified genes into different categories based on H3K4me3 and H3K27me3 enrichment around the promoter (−2500/+2500 bp regions around TSS of all protein coding genes) using SICER for calling significant peaks of enrichment (Fig. 2b-c). TSS-regions that have overlapping H3K4me3 and H3K27me3 peaks in the −2500/+2500 bp were classified as bivalent promoters [8–10, 26], (schematically shown in Fig. 2b) while regions that carry only one mark individually were classified as H3K4me3-exclusive or H3K27me3-exclusive genes. The size distribution shows that the H3K4me3 peaks are narrower (~1–4 Kb) than the H3K27me3 peaks (~4–8 Kb), while the overlapping zones are of much smaller size range (~0.5–2 Kb) (Fig. 2c). We then characterized the distribution of the peak centers relative to the TSS in these three promoter classes. The H3K4me3 peak calls are centered around the TSS in both the H3K4me3-exclusive and bivalent promoters, whereas the H3K27me3 peak calls tend to be distributed further up- or downstream from the TSS in the H3K27me3-exclusive promoters (Additional file 3: Figure S3E–G). The regions of exact overlaps of H3K4me3 and H3K27me3 peaks, which defines bivalent zones, although present around the TSS are mostly towards the upstream region (Additional file 3: Figure S3G), indicating that the polycomb mark is more upstream at bivalent promoters, unlike H3K4me3 peaks which focally occupy the TSS region.

To get an insight into the genomic position of bivalent nucleosomes, the sequencing reads binned at 10 bp intervals across the −/+5 Kb region around the TSS were plotted after averaging and normalizing to the average input reads for each of the promoter classes. As expected, H3K4me3-exclusive promoters show a general enrichment of this mark on either side of the TSS [27] with partial depletion approaching the TSS (black line, Fig. 2d; gene level plots shown as heatmaps, Additional file 4: Figure S4A) corresponding to the nucleosome free region [28, 29]. The nucleosome free zone is highlighted in the input sequencing data, which shows a decrease especially in the TSS downstream region (upto ~800 bp) relative to the upstream region in the H3K4me3-exclusive and bivalent promoters indicating more MNase accessibility and therefore a relatively open promoter configuration (Additional file 3: Figure S3H). H3K27me3-exclusive promoters on the other hand, are broadly marked by H3K27me3 across the whole TSS-region (Fig. 2e, Additional file 4: Figure S4E) without the pronounced nucleosome free zone (Additional file 3: Figure S3H), displaying a closed chromatin configuration in agreement with their low expression state (Fig. 2h) [29]. Further, although typical H3K4me3 peaks about the TSS of bivalent promoters (green line, Fig. 2d), interestingly H3K27me3 marks a much broader region with increased asymmetric enrichment ~ 2000 bp up- and down-stream from the TSS (green line, Fig. 2e). Interestingly, this asymmetric bimodal H3K27me3 distribution is unique to bivalent promoters compared to H3K27me3-exclusive promoters (green vs. red, Fig. 2e). Thus in summary, H3K27me3 enrichment at bivalent promoters show a pattern distinct from the broad distribution observed in the H3K27me3-exclusive genes.

The positions of individual bivalent mononucleosomes, mapped by sequential-ChIP sequencing reads, reveal several distinct features compared to the individual marks (Fig. 2f and Additional file 4: Figure S4I–K). Firstly, as expected from peak overlaps, the bivalent nucleosomes are maximally populated at promoters classified as bivalent (green line, Fig. 2f), but with a pattern of focal enrichment covering the TSS (lacking the characteristic dip seen for H3K4me3). This pattern is interestingly different from the zones of overlap between H3K4me3 and H3K27me3 peaks which tend to be in the upstream TSS region (Additional file 3: Figure S3G). Further, the pattern of bivalent nucleosomes in Fig. 2f is distinct both from the H3K4me3 pattern (green line, Fig. 2d), and the asymmetric distribution of H3K27me3 pattern (green line, Fig. 2e). This difference in averages pans out in the heat map patterns as well, reiterating a distinct population of bivalent nucleosomes (Additional file 4: Figure S4K). Interestingly, a lower level of bivalent nucleosomes are observed at H3K4me3-exclusive promoters showing a differential pattern compared to bivalent genes (black vs. green line, Fig. 2f). Finally, H3K27me3-exclusive promoters have no enrichment of bivalent mononucleosomes (red line, Fig. 2f). H2A.Z recapitulates the above pattern i.e. enriched at both active (H3K4me3) and poised promoters (bivalent) and absent at H3K27me3 exclusive promoters (Fig. 2g). In summary, the enrichment pattern of bivalent nucleosomes in Fig. 2f is suggestive of polycomb targeting in conjunction with trithorax at the more accessible chromatin region around the TSS in both the bivalent as well as H3K4me3-exclusive promoters [30, 31]. While individual genes may display a more heterogeneous and punctate distribution of bivalent nucleosomes, global patterns reveal their highest enrichment overlapping the nucleosome-free zone encompassing the TSS. Both the heterogeneity observed in individual genes and the global patterns of bivalent nucleosomes indicate that not all nucleosomes in a promoter with zonal bivalency are truly bivalent.

### Relationship of H3K4me3, H3K27me3, and bivalent nucleosomes with promoter CpG content

**Fig. 3:**
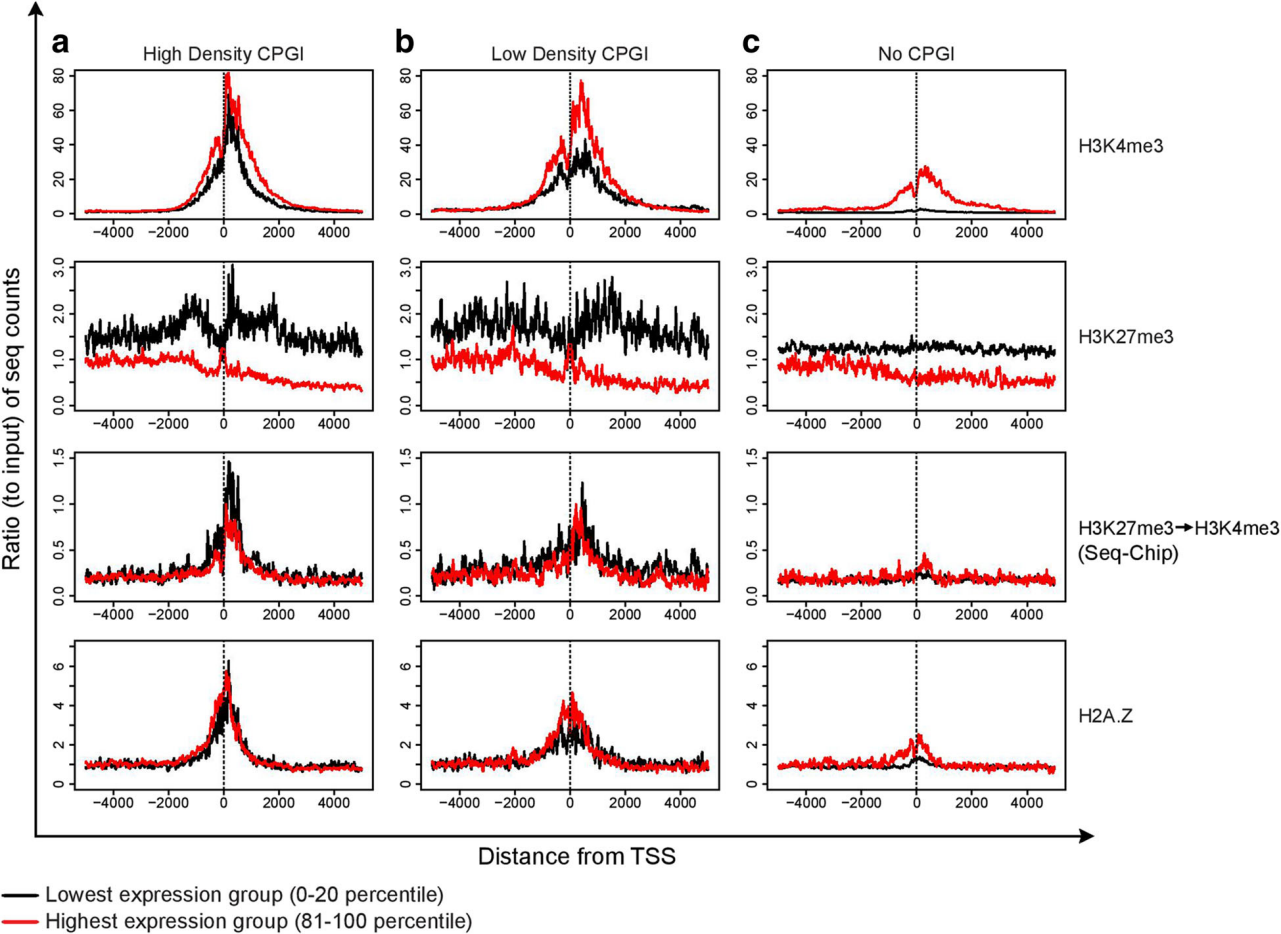
Distribution of ChIP-seq reads around the TSS for gene promoters of different CpG density. Genes were divided into different groups based on the presence or absence of a promoter-associated CpG island. CpG-island genes were further sub-classified by CpG density into quintiles, with the highest CpG density (81–100 percentile, **a**), and lowest density (0–20 percentile, **b**) CpG-island genes shown. Gene promoters without CpG islands are shown in **c**. For each plot, genes were additionally characterized for gene expression, with genes belonging to lowest (0–20 percentile) or highest (81–100 percentile) expression depicted in black and red, respectively. ChIP-seq reads were binned at 10 bp intervals. Y-axis represents average ChIP-seq read profiles normalized to corresponding average of the input. 1^st^ row, H3K4me3 ChIP-seq reads plotted as outlined above. 2^nd^ row, H3K27me3 ChIP-seq reads. 3^rd^ row, Sequential ChIP (H3K27me3 → H3K4me3)-seq reads. 4^th^ row, H2A.Z ChIP-seq reads

To further understand the relationship between CpG islands, gene expression, and positions of bivalent nucleosomes, we classified the promoters into quintiles (20 percentile intervals) of increasing CpG densities and contrasted these with genes having no promoter CpG-island. Within each such group based on CpG content, genes were further sub-classified into quintiles by gene expression levels (highest quintile in red and lowest quintile in black, Fig. 3). As expected, H3K4me3 enrichment around the TSS is proportional to the level of gene expression, but notably also increases with increasing CpG density (1^st^ row, Fig. 3a–c), indicating that the level of trithorax activity is correlated with CpG density [32]. Further, H3K27me3 enrichment is inversely proportional to the expression level of genes in all three cases (2^nd^ row, Fig. 3a–c). However, not only is this H3K27me3 enrichment reminiscent of the pattern at bivalent promoters detailed earlier (green line, Fig. 2f), this asymmetric bimodal distribution around the TSS also seems to be CpG dependent (black line, 2^nd^ row, Fig. 3a-b). This stark contrast is evident with the non-CpG-island promoters where H3K27me3 is uniformly distributed across the TSS (black line, 2^nd^ row, Fig. 3c), similar to genes classified as H3K27me3-exclusive in Fig. 2e (red line). Perhaps the most significant observation is the clear dependence of the sequential-ChIP reads (bivalent nucleosomes) on CpG islands when contrasted with non-CpG island genes (3^rd^ panel, Fig. 3a, B versus C), with only subtle differences between high- and low-density CpG island genes. Lastly, we also observe H2A.Z enrichment near the TSS with positive correlations to both CpG density as well as transcription status (4^th^ row, Fig. 3a–c). This also happens to be the region where others have mapped peaks for the highly transcriptionally correlated histone variant H3.3 [33], and assigned this histone to salt labile nucleosomes [16]. When put in perspective, our results summate possibility of nucleosomes at the TSS proximal open chromatin regions being marked by both, bivalency as well as H2A.Z, specifically in CpG island containing genes alone, and having a distribution that is correlated with transcription status.

In addition to the promoter regions, we further analyzed the presence of bivalent nucleosomes at enhancer elements (ChromHMM defined enhancers from the ENCODE project, subtracted for promoters), which are not only associated with a myriad of chromatin marks [34], including H3K4me3 and H3K27me3 but have also been shown to be associated with labile nucleosomes [16, 35]. In comparison to a random set of genomic elements (of the same size distribution), enhancer regions show a clear enrichment of H3K4me3 and H2A.Z, but no significant enrichment of H3K27me3 and bivalent nucleosomes (Additional file 5: Figure S5, 1^st^ and 2^nd^ panel). The chromatin profile of the enhancer regions when divided into subsets based on the presence or absence of a CpG islands however, reveal that enhancers with CpG island (s) have elevated H3K27me3 and bivalent nucleosomes, as well as increased H2A.Z (Additional file 5; Figure S5, 3^rd^ and 4^th^ panel). Thus, like the promoter regions, presence of bivalent nucleosomes is again correlated to presence of CpG islands.

### Bivalent nucleosomes and CpG methylation do not co-exist

**Fig. 4:**
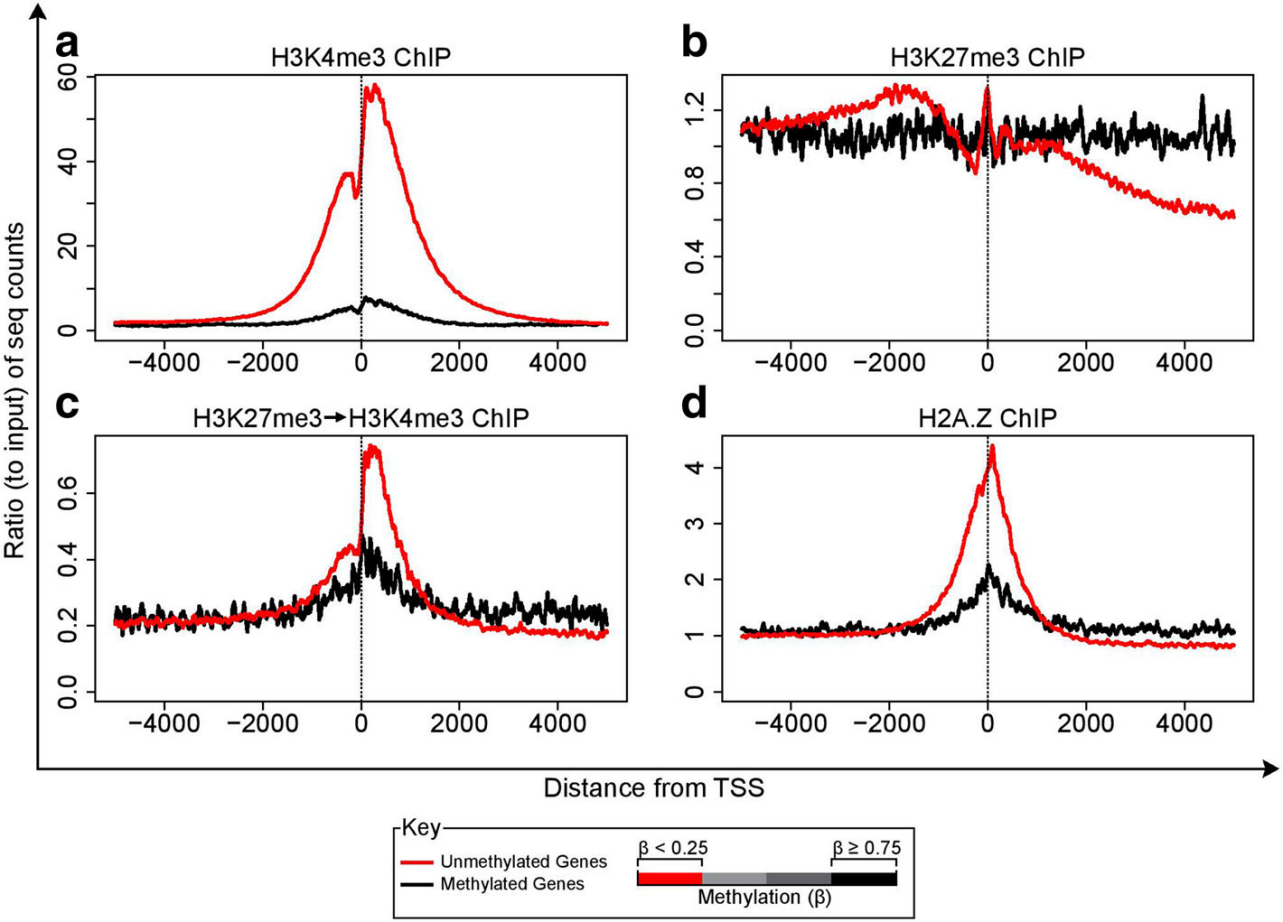
Distribution of ChIP-seq reads around the TSS for gene promoters that are DNA-hypermethylated or unmethylated. Gene promoters were identified as methylated or unmethylated based on the Infinium methylation array data. H3K4me3 **a** H3K27me3 **b** sequential-ChIP **c** and H2A.Z **d** ChIP-seq reads were binned at 10 bp intervals and the average ChIP-seq reads normalized to corresponding average of the input plotted (Y-axis)

Given the prevalence of bivalent nucleosomes in CpG islands and the known relationship between bivalency in ESC and later DNA hypermethylation in cancer cells, we examined the association of our bivalent nucleosomes with hypermethylated promoters. Previous data from our lab [10] has shown that regions with bivalent promoters in the embryonic and adult stem cell setting make up an inordinate percentage of genes which show aberrant cancer-specific DNA methylation changes. Accordingly, we used the Illumina DNA methylation array platform to separate CpG island genes into least methylated (β < 0.25) and hypermethylated (β > 0.75) genes and analyzed the histone marks in these landscapes (Fig. 4). In contrast to the above data highlighting the association of bivalent nucleosomes with the presence of promoter CpG islands (Fig. 3), when DNA hypermethylated (black line, Fig. 4), such promoters not only lack the bimodal distribution of H3K27me3 enrichment but are almost devoid of both H3K4me3 and bivalent nucleosomes (Fig. 4a–c, black lines). Similarly, H2A.Z preferentially occupies the region immediately surrounding the TSS in unmethylated promoters (Fig. 4d, red line), as reported earlier [36]. Put together, these data indicate that closed chromatin associated with DNA hypermethylation at promoter CpG islands, and their virtual lack of transcriptional capacity [37], excludes the presence of both the trithorax and polycomb marks individually and in combination as bivalent mononucleosomes.

## Discussion

To deepen our understanding of how seemingly opposing chromatin modifications combine to alter gene expression, we report the first genome-wide positional mapping of nucleosomes with H3K4me3 and H3K27me3 marks. We performed modified sequential-ChIP on purified mononucleosomes followed by next-generation sequencing to map the positions of individual bivalent nucleosomes relative to the distribution of the individual H3K4me3 and H3K27me3 marks that constitute zonal bivalency. Further, we analyzed the relationship of bivalent mononucleosomes with gene expression, promoter CpG content and DNA methylation. The idea that combinatorial marks can coexist on the same nucleosome is important for deciphering the roles of histone modifications and their implications for control of gene expression [4, 5]. The methods described here will allow exploration of other combinatorial histone modifications such as H3K27me3 and H3K36me2/3 marks [15, 25, 38].

The concept of bivalent chromatin consisting of opposing H3K4me3 and H3K27me3 modifications is thought to help maintain genes in a low but poised state of expression in stem cells [7–9, 39]. Virtually all studies thus far have identified bivalent domains from linear maps of H3K4me3 and H3K27me3 overlaps rather than at nucleosome resolution. In contrast, the question of co-existence of the marks on the same nucleosome has been less explored, at least in part due to technical difficulties of mapping at nucleosome resolution. In initial studies, sequential ChIP of multinucleosome substrates was used for this purpose and, despite the caveats outlined previously, it was suggested that individual bivalent nucleosomes exist [8, 26]. A more recent proteomic approach using mass spectrometry on purified histone H3 tail peptides, demonstrate bivalent nucleosomes carrying both marks on opposite peptide tails [15], in agreement with our findings here (Fig. 1i). However, candidate gene studies so far have analyzed true bivalent nucleosomes only at few gene promoters, and thus very little is known regarding global positioning of bivalent nucleosomes and their frequency.

Our findings stress the need to consider chromatin bivalency in two contexts—namely the zonal patterns first identified [8], but also the existence and positioning of individual bivalent mononucleosomes. First, in relation to zonal patterns of bivalency, we observe that the zones where H3K4me3 and H3K27me3 generally tend to overlap are upstream to the TSS (Additional file 3: Figure S3G), whereas the sequential-ChIP data shows that the bivalent mononucleosomes enrich immediately around the TSS (Figs. 2, 3 and 4). Second, although the H3K4me3 peaks are prominent (green line, Fig. 2d), we observe a decrease of H3K27me3 immediately flanking the TSS of promoters deemed bivalent (green vs. red line, Fig. 2e). These observations tend to suggest a model where Polycomb Repressive Complex 2 (PRC2) is recruited uniformly across broad regions around promoters but antagonized around the TSS of CpG-island promoters where trithorax proteins show maximal activity of establishing the H3K4me3 modification, even at promoters that have low activity (Fig. 3). Such an epistatic relationship is also supported by observations that methylation of H3K27 by PRC2 is inhibited by nucleosome substrates carrying H3K4me3 or H3K36me3 on both H3 tails of the octamer (symmetric modification) but not when only a single H3 tail is modified asymmetrically [15]. Thus, although zones of bivalent chromatin may be in part the result of heterogeneous cell populations that carry one or the other mark, the combined targeting of trithorax and polycomb proteins results in a focal enrichment of bivalent nucleosomes just around the TSS (Fig. 2f).

With regards to true bivalent mononucleosomes, our current findings challenge some previous studies and conclusions, probably due to alterations in the way we extract nucleosomes to best preserve the salt labile fractions of chromatin. Others have shown that PRC2 components are enriched around promoters [40, 41] with CpG-islands, and that this state correlates with absence of transcription factor (TF) binding sites [31]. These studies indicate that H3K27me3, and any form of bivalency, should be completely absent from the active promoters. However, we do observe a focal enrichment of bivalent nucleosomes in the immediate vicinity of the TSS in genes with promoter CpG islands, biased to the downstream open chromatin region and even for active genes (Figs. 2f and 3). These data indicate that in active genes, as well as in bivalent genes with generally lower basal activity, the chromatin immediately surrounding the TSS is accessible to and/or recruit PRC2. This feature of PRC2 recruitment at active promoters might be crucial in the transient and immediate shutdown of transcription by PRC components during acute DNA damage [42].

The observed enrichment of bivalent nucleosomes at active genes could be due to non-specific flow-through of H3K4me3 in the primary ChIP with anti-H3K27me3, and subsequent enrichment in the secondary ChIP with anti-H3K4me3 antibody. However, the ChIP-PCR data in Fig. 1e, g shows that there is very little flow-through of H3K4me3-nucleosomes in the primary ChIP reaction. Further, we addressed this by analyzing the raw reads obtained in the sequential ChIP-seq and H3K4me3 ChIP-seq for gene sets sub-grouped into quintiles of decreasing expression levels (Additional file 6: Figure S6). Although H3K4me3 levels positively correlates with expression, the sequential ChIP-seq reads do not correlate with H3K4me3 enrichment pattern, indicating that the sequential ChIP-seq reads obtained here are not just a flow-through of H3K4me3 nucleosomes. On a technical note, due to the scarcity of sequential ChIP-seq reads giving rise to sparse reads distributed across the genome, using paired-end reads will further help reduce the noise.

With respect to individual bivalent nucleosomes, perhaps the most important and potentially functional aspect of our findings is their relationship to the TSS. For both, genes classified as carrying zonal bivalency and those which are predominantly marked by H3K4me3, there exists a strong correlation between bivalent mononucleosomes and the so-called “nucleosome-free region”. Strikingly, this region is known to harbor salt-labile nucleosomes that contain at least two important non-canonical histone variants: H3.3 and H2A.Z, which can be incorporated and evicted from chromatin in a DNA replication-independent manner [16, 43]. These nucleosomes are thought to play a key role in fostering transcription, or potential for activation thereof. Combining our analysis of global H2A.Z positioning with the positioning of bivalent mononucleosomes strongly indicates that at least a subset of these exist as non-canonical H2A.Z-containing nucleosome core particles. Given that H2A.Z facilitates association of both PRC2 and MLL at promoters [44], it is perhaps unsurprising that we should observe co-localization of bivalent nucleosomes with H2A.Z. Similarly, deposition of H3.3 at promoters is known to be required for proper establishment of bivalency [45]. However, H2A.Z and H3.3 themselves appear to exert opposing forces on chromatin structure and transcription [46], and the precise relationship between these non-canonical nucleosomes and bivalency has as yet not been fully characterized. Our data highlights the interplay between several opposing marks that must be resolved in ESCs upon lineage commitment.

Our data adds to the growing body of work examining the relationship between localization of bivalency and CpG island/density (Fig. 3), as previously suggested both by comparative analysis [47] and synthetic CpG designs [48], and furthers our understanding towards cancer specific DNA methylation. Many of the genes abnormally methylated in cancer are those which are normally marked by zonal bivalency in ES cells [10]. In this normal context, bivalency has been associated with low expression of this key set of genes, which may help to maintain the cells in an undifferentiated state [10]. Our observation that bivalent mononucleosomes reside exclusively in promoters of CpG island genes, in both active and poised states indicates that such genes generally have some degree of open chromatin proximal to the TSS. In contrast, closed chromatin associated with DNA hypermethylation appears to preclude the presence of individual bivalent nucleosomes. This mutual exclusion between DNA methylation of promoter CpG islands and bivalent mononucleosomes are in agreement with previous observations of exclusion of both MLL and PRC2 by methylated DNA, and the loss of H2A.Z from hypermethylated promoters [3, 36].

## Conclusions

In summary, to the best of our knowledge, this study is the first attempt to map combinatorial histone modification maps at a genome wide level using mononucleosome substrates. Our findings refine the concept of bivalent chromatin and further classify bivalency into two different yet associated subcategories of zonal bivalency and individual mononucleosome bivalency. In further studies, the relationship between these two types of bivalency and the potential roles of each in gene regulation must be considered. Not only should the techniques developed herein be instrumental in fostering genome-wide mapping of combinatorial marks, but the results open doors to studies of functions for such patterns and particularly those for truly bivalent mononucleosomes in genome positioning and the control of gene transcription.

## Methods

### Cell culture and harvesting

SW480, HCT116 and DKO cells were grown in McCoy’s 5a (Corning) while RKO cells were grown in MEM (Corning) supplemented respectively with 10 % FBS (Gemini). NCCIT cells were grown in RPMI (Corning) with 15 % FBS. All cells were grown in 5 % CO_2_ at 37 °C to 70–80 % confluence in 150 mm culture dishes. Cells were pre-chilled on ice, harvested by scraping, and washed once with chilled PBS with freshly added 1X complete proteinase inhibitor (Roche) and 1 mM AEBSF. The pellet volume was measured by weight and stored at −80 °C until further use.

### Mononucleosome preparation

All preparations were carried out in buffers with freshly added proteinase inhibitor cocktail and 1 mM AEBSF maintained at 4 °C/on ice unless mentioned otherwise. Each single ChIP experiment was performed from 1X volume of nuclei isolated from 6 million cells as previously described [42], followed by washing 5 volumes of 1X MNase reaction buffer (50 mM Tris-HCl [pH 7.6], 4 mM MgCl_2_, 2 mM CaCl_2_) and re-suspended in the 3 volumes of the same buffer containing 0.2 % Triton X-100. Samples were treated with 3 U/ml of MNase for 10 min at 37 °C followed by mild sonication to release the digested nucleosomes. Reactions were stopped by addition of 30X stop buffer (300 mM EDTA [pH 8.0], 150 mM EGTA [pH 8.0]). Chromatin was cleared by centrifugation at 13,000 × *g* for 20 min at 4 °C, and the supernatant was crosslinked with 0.15 % formaldehyde (reaction stopped by 50 mM glycine) followed by repetition of the centrifugation step. The supernatant was further fractionated in a 5–25 % sucrose gradient (S.G.) prepared in low salt ChIP buffer (20 mM Tris-HCl [pH 8.0], 20 mM NaCl, 2 mM EDTA, 0.2 % Triton X-100, 0.1 % SDS) and separated at 40,000 rpm for 16 h in a Beckman SW40 Ti rotor. Pooled mononucleosome peak fractions were exchanged for 1X ChIP buffer (0.1 % SDS, 1 % Triton 2 mM EDTA, 20 mM Tris-HCl [pH 8.0], 150 mM NaCl) using PD10 desalting columns (GE Healthcare) and adjusted to 10 μg/ml to be used for ChIP.

Alternatively, for “quick prep protocol” (Q.P.), nuclear pellets were washed (minus detergent) and resuspended in one volume of 2X MNase reaction buffer (100 mM Tris-HCl [pH 7.6], 8 mM MgCl_2_, 4 mM CaCl_2_, 0.4 % Triton X-100) on ice, followed by treatment with 8 U/ml of MNase at 37 °C for 10 min shaking at 100 rpm. The reaction was stopped with appropriate volume of 30X MNase stop buffer (300 mM EDTA [pH 8.0], 150 mM EGTA [pH 8.0]) followed by centrifugation at 13,000 × *g* for 20 min at 4 °C. Supernatant was diluted in PBS to a chromatin concentration to 200 μg/ml (A_260_) and crosslinked with 0.15 % formaldehyde for 10 min at RT before stopping the reaction with 50 mM glycine. The preparation was cleared by centrifugation at 13,000 x *g* for 20 min at 4 °C. The salt concentration from both nucleosome preps was readjusted to 1X ChIP buffer composition using appropriate stock solutions to obtain a chromatin concentration of 50 μg/ml to be used in ChIP.

### Modified chromatin immunoprecipitation

Antibodies used in the chromatin immunoprecipitation (ChIP) reactions were anti-H3K4me3 (07–473, Millipore), anti-H3K27me3 (07–449, Millipore), anti-H2A.Z (ab4174, Abcam) and anti-H3 (ab1791, Abcam). All immunoprecipitation buffers were fortified with freshly added proteinase inhibitor cocktail and 1 mM AEBSF. For each reaction of primary ChIP (1°), 5 μg of respective antibodies were bound to 40 μl of pre-blocked protein A + G Dynabeads (ratio of 4:1, Invitrogen, pre-blocked for 1 h in blocking buffer [0.5 % BSA in 1X PBS]) overnight at 4 °C, followed by two washes in PBS. Antibody-bound beads (Ab-beads) were crosslinked using 80 μM DSG at RT for 10 min, and the reaction was terminated using 50 mM glycine for 30 min followed by extensive washing of the crosslinked Ab-beads with ChIP buffer. Beads were blocked at 4 °C overnight, washed with ChIP buffer and subsequently incubated with the appropriate immunoprecipitation substrate (100 μg mononucleosomes from quick prep protocol, or 20 μg purified mononucleosomes from sucrose gradient protocol or 20 μg histone peptides) overnight at 4 °C with rotation. Samples were washed multiple times in varying conditions and combinations of buffers optimized for specificity with respect to each antibody, namely ChIP buffer, high salt ChIP buffer (0.1 % SDS, 1 % Triton X-100, 2 mM EDTA, 20 mM Tris-HCl [pH 8.0], 500 mM NaCl) and RIPA buffer (20 mM Tris-Cl [pH 7.5], 1 mM EDTA, 150 mM NaCl, 0.5 % sodium deoxycholate, 1 % IGEPAL ca630). The immunoprecipitate was then eluted in ChIP elution buffer (50 mM Tris [pH 8.0], 10 mM EDTA, 1 % SDS) at 65 °C for 10 min with agitation.

Alternatively for Sequential ChIP, five 1° ChIP reactions were eluted in 100 μl 0.5 % SDS, 200 mM β-mercaptoethanol at 55 °C for 2 min with agitation. Pooled eluted supernatants were then passed through a G25 spin column (GE Healthcare) pre-equilibrated with 1X ChIP buffer, followed by dilution of the resulting eluate with 1X ChIP buffer to about 20-fold excess. These eluates were used as substrate for secondary ChIP (2°) with a specific pre-bound antibody, bound and/or crosslinked to protein A + G Dynabeads (as detailed above) and incubated at 4 °C overnight followed by extensive washing with ChIP and RIPA buffer. The immunoprecipitate was eluted in 1X ChIP elution buffer.

Crosslinks on the eluted immunoprecipitates were reversed by overnight incubation at 65 °C. The eluted DNA was treated with 100 μg/ml RNase A (Thermo) at 37 °C for 1 h followed by Proteinase K (NEB) treatment at 20 μg/ml in a final concentration of 0.5 % SDS. The resulting DNA was cleaned up using a Qiagen PCR purification kit, and eluted in RNase DNase free 10 mM Tris pH 7.5 for further analysis. DNA obtained by ChIP was analyzed by PCR using primers shown in Additional file 7.

### Histone purification by HPLC

Uncross linked mononucleosomes prepared by the “quick prep” protocol were acid precipitated and used to purify individual histones using an Aquapore RP300 HPLC column with acetonitrile as the medium of flow, as described [23]. Peak fractions of individual histones were pooled and lyophilized. All subsequent steps were carried out at 4 °C and all buffers were supplemented with freshly added protease inhibitor cocktail. The lyophilized protein was dissolved in unfolding buffer (7 M urea, 10 mM Tris [pH 8.0], 1 mM EDTA, 0.1 M NaCl) and dialyzed overnight with high salt refolding buffer (20 mM Tris [pH 8.0], 1 mM EDTA, 1.8 M NaCl) followed by a second dialysis overnight with refolding buffer (20 mM Tris [pH 8.0], 1 mM EDTA, 150 mM NaCl). The refolded histones were clarified by centrifugation at 13,000 × *g* for 20 min at 4 °C and used as substrate for immunoprecipitation.

### Dot blot analysis

Following immunoprecipitation with respective substrates, beads were eluted directly in 1X NuPage LDS sample buffer (Life Technologies) supplemented with 20 mM β-mercaptoethanol. The eluate was then heat denatured, dot blotted onto nitrocellulose membrane (GE Healthcare) and probed with anti-H3K4me3 antibody. HRP-linked donkey anti-rabbit IgG (NA934V, GE Healthcare) was used as secondary antibody. After extensive washing, the blots were developed using SuperSignal West Pico Chemiluminescent Substrate (Thermo Scientific).

### Sequencing and mapping of chromatin immunoprecipitated (ChIP) DNA

Immunoprecipitated DNA was subjected to sequencing library preparation and sequenced on Applied Biosystems SOLiD (V3). Reads were aligned to hg18 (NCBI36) using Bioscope 1.2.1, finding all alignments between the first 25 bp of the read (seed) and the reference sequence with up to two mismatches. Each match is extended to the full length of the read, scoring 1 point for matching and −2 points for mismatching bases. The read is trimmed to the length with the highest score. If there is only one alignment or if an alignment scores significantly higher than the others for the same read, it is considered unique and reported.

### Data processing and identifying genes with enriched chromatin marks

Single end sequencing reads were processed using SAMtools [49] to remove duplicates and create BAM files of only mapped reads with mapping quality (MAPQ) better than 20. BAM file was converted to BED format using BEDTools [50], and reads with not more than one mismatch (NM is 0 or 1) were retained. Filtered data was used in all analysis.

Peaks were called using SICER [51] to detect regions enriched for the histone marks (called peaks), including broad regions. Larger gap size of 1000 bp was used to identify the H3K27me3 peaks that are typically broad. Gap size of 600 bp was used to identify the H3K4me3 peaks. Other paramters used are: Redundancy Threshold =1, Window Size = 200 bp, Fragment Size = 200 bp, Effective Genome Fraction = 0.75. Peaks were identified at a false discovery rate (FDR) of > = 0.01. Gene annotation data (protein coding genes) was downloaded from BioMart (may2009.archive.ensembl.org), and genes that had peak (s) within +/−5000 bp from their TSS were called “enriched” for that mark at the promoter. Promoters that had H3K4me3 and H3K27me3 peaks overlapping in this region were called bivalent promoters, and the remaining promoters were classified as H3K4me3-exclusive or H3K27me3-exclusive, or ‘none’ for genes with none of the marks.

### Analysis of CpG-island and enhancers

CpG island data was downloaded from UCSC (hg18_cpgIslandExt). Promoters with CpG islands were identified as promoters that have a CpG-island within +/−1500 bp from the transcription start site (TSS). Genes with CpG-islands were sub settled into 5 groups (quintiles, 0–20, 21–40, 41–60, 61–80, 81–100) based on increasing CpG-density as defined in the CpG-island data from UCSC. Gene promotes that did not match any CpG-island were called non-CpG-island promoters. Alternate annotated transcripts of a gene, for which any of the other TSS had a CpG-island, were removed from the analysis. Otherwise, alternate transcripts with CpG-islands were considered as individual promoter sites.

Combined genome-segmentation data (ChromHMM and Segway software) for human embryonic stem cells was downloaded from ENCODE [52]. This represents predicted enhancer regions using binding data of nine factors (chromatin marks), and open chromatin regions assessed by DNase-seq assays and a FAIRE-seq. The Refseq annotated promoter regions were removed to obtain a putative set of enhancers. A random set of 1000 enhancers was used in the analysis. Another random set of 1000 genomic elements of the same size distribution as the sampled enhancers was used to compare distribution of the chromatin marks with respect to the enhancers. Since enhancers are of different sizes, to visualize the distribution of the histone marks at enhancers, the enhancers were divided into 10 intervals and the sequencing coverage was estimated for each interval. The coverage for the 5000 bp region flanking each side of the enhancer binned at 10 bp was estimated. The profile of the whole region spanning the enhancer and +/−5000 bp flanking region was plotted after normalizing to input.

Enhancers with an overlapping CpG-island were identified as enhancers with CpG-islands. About 30 % of the sampled enhancers have CpG-islands.

### Visualization of ChIP-seq data—individual genes and profile plots

The Integrative Genomics Viewer (IGV) browser was used to display ChIP-seq data [53]. For the average profile plots, the coverage of the ChIP-seq reads were estimated at 10 bp bins using BEDTools in a region spanning 5000 bp upstream and downstream from the TSS. We also tried computing coverage in 200 bp bins for the average plots, but due to the loss of resolution while averaging the pileup of reads, the 10 bp bins were selected. Promoter regions were aligned at the TSS with direction of transcription form left to right. The profile plots were generated as ratio to the average input sequencing reads for each category of promoters analyzed. In the plots, the normalized values for sequential-ChIP-seq data (Bivalent mononucleosomes) is below 1, even at enriched regions, because sequential-ChIP yields very low amount of DNA corresponding mononucleosomes marked with both marks and background DNA, which then is amplified resulting in reads that are multiplicated compared to reads for direct ChIP-seq and input-seq. We removed multiplicated reads to avoid any bias. Hence, the sequential-ChIP-seq reads are far lower compared to input, which results in ratios below 1 at all positions.

### Gene expression and DNA methylation arrays

RNA was extracted from the NCCIT cells and processed for hybridization on an Agilent 4 × 44 K array as described previously [54]. The mock channels were extracted and quantile-normalized using the R statistical computing platform and limma package from the Bioconductor bioinformatics software project (http://bioinf.wehi.edu.au/limma/). The log2 intensities of probes were used for plotting.

For genome-wide DNA-hypermethylation analysis, the Infinium methylation array (27 k) was used to analyze bisulfite-treated DNA (EZ DNA-Hypermethylation Kit, Zymo Research) as per standard protocols [18]. β-Values were computed as the signal of the methylation-specific probe over the sum of the signals of the methylation- and unmethylated-specific probes. Probes with poor signals (*P*-value > 0.05) were not considered. Promoters with at least one probe with β-Value ≥ 0.75 were considered as methylated promoters while those with < 0.25 were considered as unmethylated.

### Computational tools and software

All genomic analysis was done in the R computing platform using Bioconductor packages. Plots were further annotated in Adobe Illustrator.

## Additional files

Additional file 1: Figure S1. Sucrose gradient purification and analysis of mononucleosomes from NCCIT. MNase treated chromatin was fractionated by a 5–25 % sucrose gradient and analyzed by agarose gel electrophoresis to identify fractions bearing mononucleosomes, (Mononucleosome peak fractions are 16 to 22 as indicated by the numbers). (JPG 234 kb) Additional file 2: Figure S2. Standardization of sequential ChIP. A, Schematic representation of the Sequential ChIP protocol (Seq-ChIP) using DSG crosslinker to tether the primary antibody to the protein A + G beads, allowing efficient elution by SDS thereby avoiding carryover in the subsequent IP. B, Western blot analysis of efficacy of antibody cross-linking to beads by DSG, as monitored by subsequent elution with SDS. C, Optimized conditions of Seq-ChIP using SW480 mononucleosomes (Q.P.) as substrate, tested by PCR (MLH1, primer set-B). Gel analysis comparing standard single antibody mediated ChIPs (single) versus sequential IPs (eg. 1° ChIP using anti H3 antibody followed by 2° ChIP using normal rabbit IgG is depicted as H3 → IgG), where H3-1° → H3-2° serves as the positive test while H3 → IgG as the negative control. (JPG 305 kb) Additional file 3: Figure S3. Distribution of bivalent nucleosomes, zonal-bivalency and nucleosome-free region around the TSS of the various promoter classes. A–D, Distribution of bivalent nucleosomes at candidate gene promoters. E–G, Distribution of the H3K4me3, H3K27me3 peak calls, and their overlapping regions, that were used to classify genes into H3K4Me3-exclusive and H3K27me3-exclusive promoters. Genes that have a H3K4me3 or H3K27me3 peak call within +/−2500 bp from the TSS were identified, and the gene promoters regions were parsed into those that have exclusively H3K4me3 peaks (H3K4me3-exclusive), exclusively H3K27me3 peaks (H3K27Me3-exclusive) and those with overlapping H3K4me3 and H3K27me3 (bivalent). The distances of the center of the peak calls and overlapping regions from the TSS are plotted in E, F and G. The distribution of the distances from the TSS is plotted to depict the probability of occurrence of the peaks at varying distances from the TSS (x-axis, 0 position) for H3K4me3-exclusive promoters (E), H3K27Me3-exclusive promoters (F) and bivalent promoters (G). H, Top panel: Input sequencing reads to show the nucleosome-free zone. Bottom panel shows sequencing reads downstream from the TSS relative to the symmetric position upstream from the TSS as Log2 ratios (y-axis). Depletion in sequencing reads in the 0–1000 bp region for H3K4Me3-exclusive (black) and bivalent promoters (green) is more prominent compared to the H3K27me3-exclusive (red) promoters and promoters marked with none (blue) of the marks analyzed here. (JPG 547 kb) Additional file 4: Figure S4. Heat maps comparison of single ChIP versus sequential ChIP data. Gene level plots as heatmaps showing input normalized ChIP-seq reads in +/− 2.5Kb window around the TSS. Genes in each panel is ordered according to the increasing read counts (top to bottom) of the Sequential ChIP (H3K27me3 → H3K4me3)-seq reads for each category of promoters (from Fig. 2h, labeled to the left of each row). Scale bar shows color scale of normalized read counts. (JPG 717 kb) Additional file 5: Figure S5. Analysis of enhancer regions based on CpG islands. Distribution of the H3K4me3, H3K27me3, bivalent and H2A.Z mononucleosomes at enhancers and flanking regions. ChromHMM defined enhancers in human embryonic stem cells (ENCODE project) was downloaded from UCSC and subtracted for Refseq promoter regions. Average profile of the input normalized sequencing coverage data for a random set of 1000 genomic elements (1st row) and 1000 enhancer elements (2nd row), divided into 10 intervals, and their flanking regions (detailed in methods) are plotted. 3rd and 4th row show the profiles for the enhancer elements subsetted, respectively, by presence or absence of CpG-island (s) at the enhancers. Plots on the right column are identical to those on the left except that the y-axis is zoomed to show the typically lower enrichment of H3K27, H2A.Z and bivalent nucleosomes (compared to H3K4me3). (JPG 581 kb) Additional file 6: Figure S6. Distribution of raw ChIP-seq reads for H3K4me3, H3K27me3 and sequential ChIP-seq. Average of raw reads from H3K4me3 ChIP-seq (A), H3K27me3 ChIP-seq (B) and Sequential ChIP (H3K27me3 → H3K4me3)-seq (Seq-ChIP-seq) reads (C) for the groups of genes sub-setted into quintiles (0–20, 21–40, 41–60, 61–80, 81–100) of increasing expression levels (obtained from gene expression microarray data) as shown in color key. The Seq-ChIP-seq reads for the different expression quintiles are not correlated with that of the H3K4me3 enrichment, which shows a decreasing enrichment as the gene expression levels decrease. The lack of correlation between H3K4me3 and Seq-ChIP-seq reads for the different gene expression quintiles indicate that the Seq-ChIP-seq reads are not non-specific flow-through of H3K4me3 in the primary ChIP with anti-H3K27me3. (JPG 380 kb) Additional file 7: Supplemental Methods. (DOCX 15 kb)

## Abbreviations

DSG: Disuccinimidyl glutarate
ESC: Embryonic stem cell
H3K27me3: Histone H3 lysine 27 trimethylation
H3K27me3: Histone H3 lysine 4 trimethylation
MLL: Mixed lineage leukemia
MNase: Micrococcal nuclease
PRC: Polycomb repressive complex
seq-ChIP: Sequential ChIP
TSS: Transcription start site
