# Supplementary figures and images for: Genome-wide positioning of bivalent mononucleosomes
Source: BMC Med Genomics. 2016 Sep 15;9:60. doi: 10.1186/s12920-016-0221-6 (PMC5025636; doi:10.1186/s12920-016-0221-6)

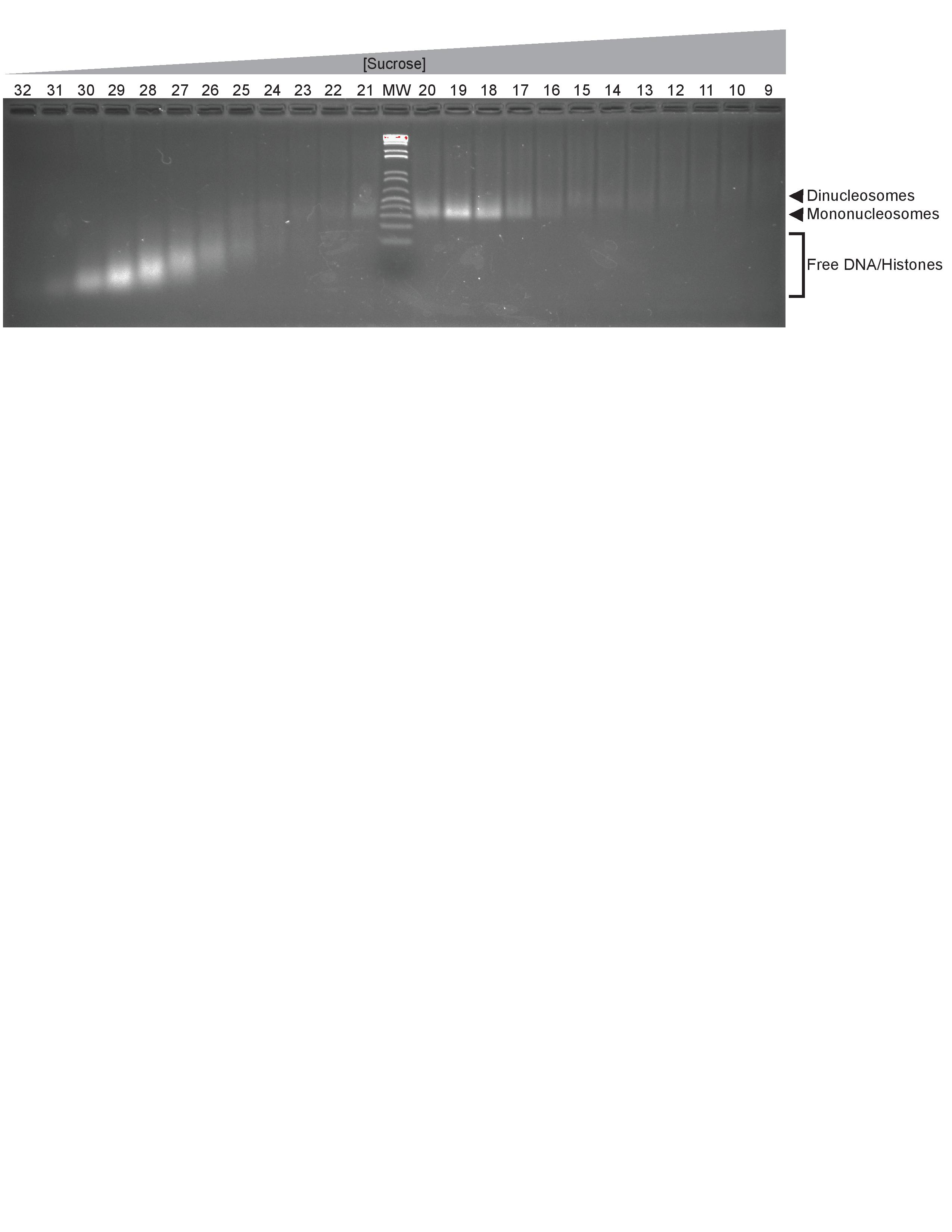

Supplement: Additional file 1: Figure S1. — Sucrose gradient purification and analysis of mononucleosomes from NCCIT. MNase treated chromatin was fractionated by a 5–25 % sucrose gradient and analyzed by agarose gel electrophoresis to identify fractions bearing mononucleosomes, (Mononucleosome peak fractions are 16 to 22 as indicated by the numbers). (JPG 234 kb) [file 12920_2016_221_MOESM1_ESM.jpg]

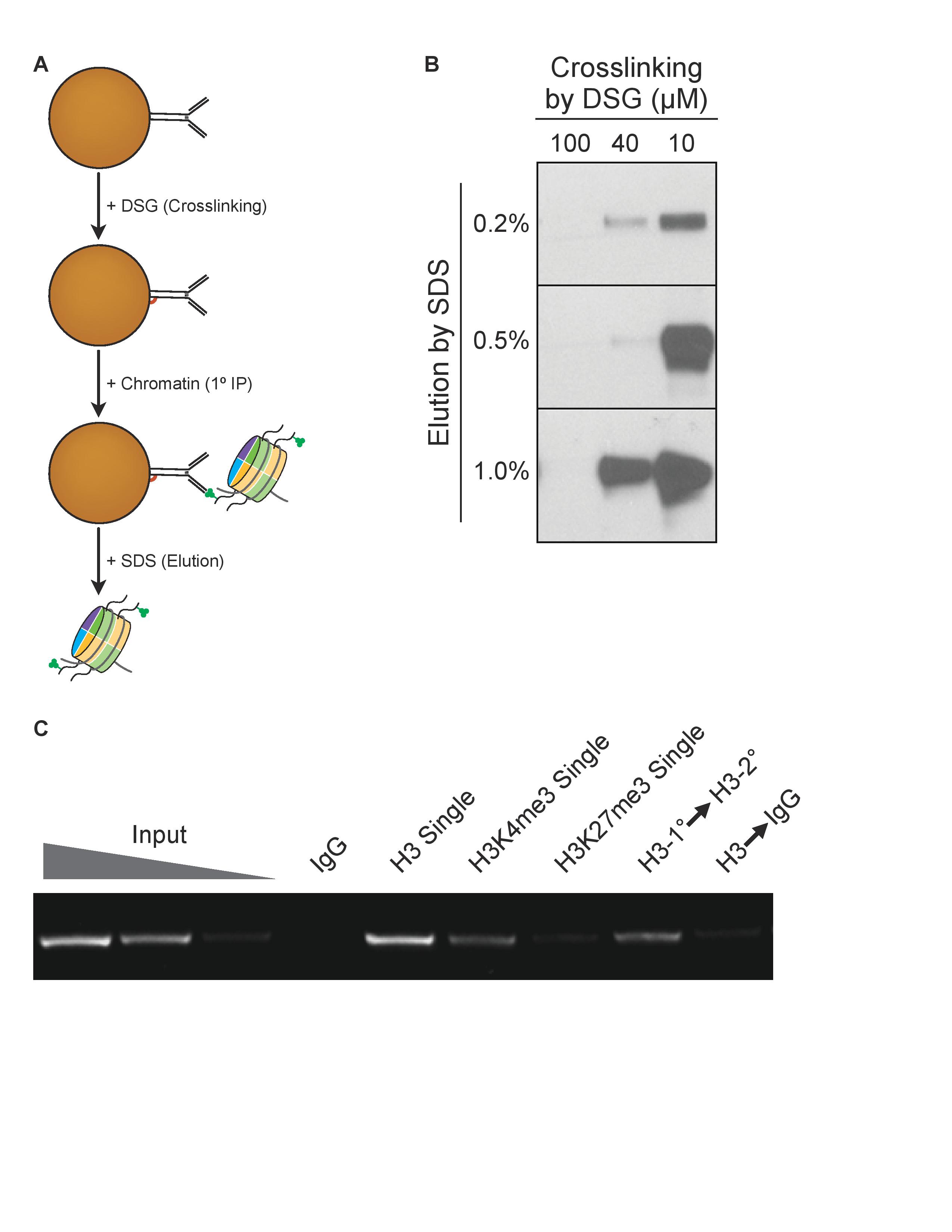

Supplement: Additional file 2: Figure S2. — Standardization of sequential ChIP. A, Schematic representation of the Sequential ChIP protocol (Seq-ChIP) using DSG crosslinker to tether the primary antibody to the protein A + G beads, allowing efficient elution by SDS thereby avoiding carryover in the subsequent IP. B, Western blot analysis of efficacy of antibody cross-linking to beads by DSG, as monitored by subsequent elution with SDS. C, Optimized conditions of Seq-ChIP using SW480 mononucleosomes (Q.P.) as substrate, tested by PCR (MLH1, primer set-B). Gel analysis comparing standard single antibody mediated ChIPs (single) versus sequential IPs (eg. 1° ChIP using anti H3 antibody followed by 2° ChIP using normal rabbit IgG is depicted as H3 → IgG), where H3-1° → H3-2° serves as the positive test while H3 → IgG as the negative control. (JPG 305 kb) [file 12920_2016_221_MOESM2_ESM.jpg]

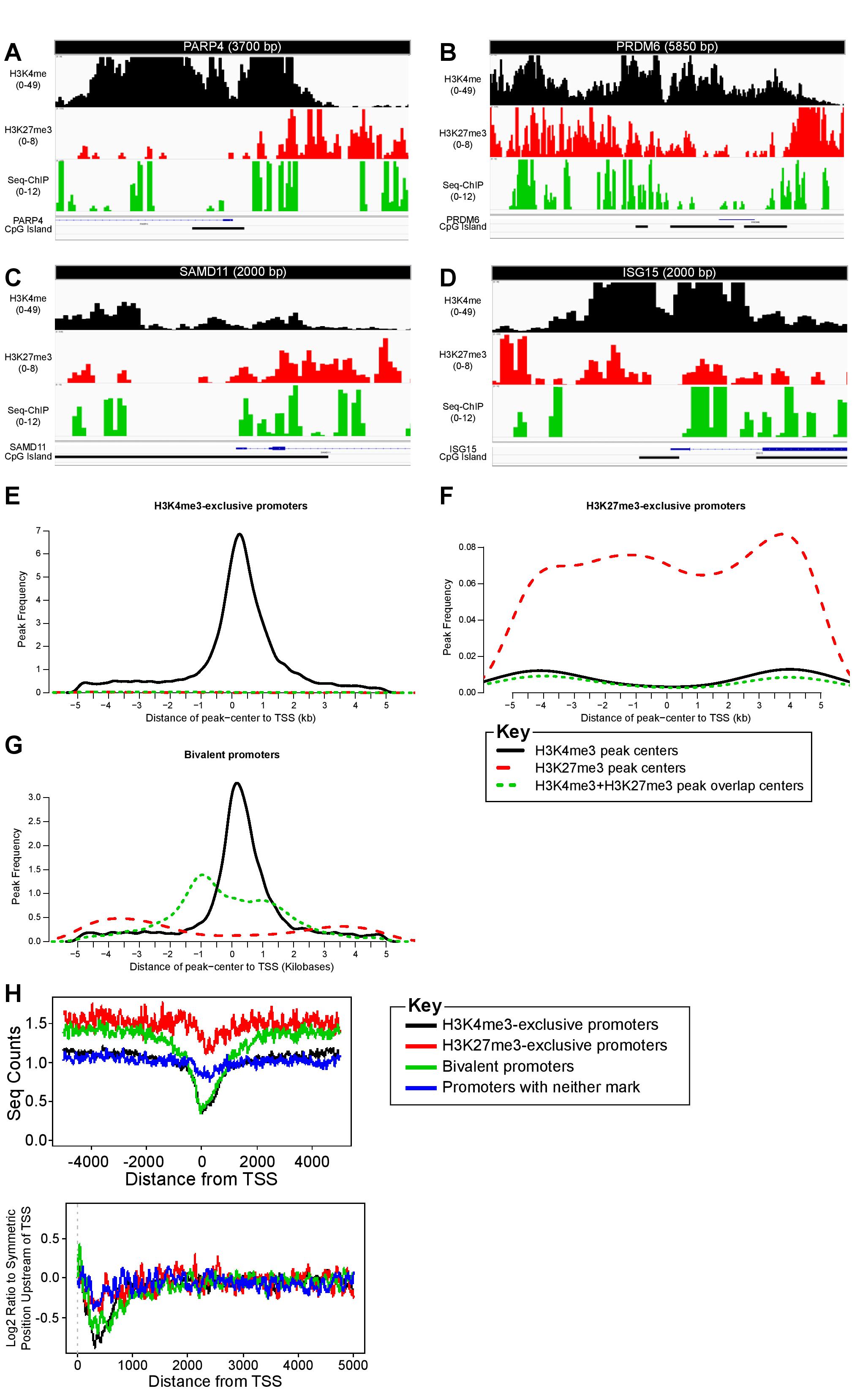

Supplement: Additional file 3: Figure S3. — Distribution of bivalent nucleosomes, zonal-bivalency and nucleosome-free region around the TSS of the various promoter classes. A–D, Distribution of bivalent nucleosomes at candidate gene promoters. E–G, Distribution of the H3K4me3, H3K27me3 peak calls, and their overlapping regions, that were used to classify genes into H3K4Me3-exclusive and H3K27me3-exclusive promoters. Genes that have a H3K4me3 or H3K27me3 peak call within +/−2500 bp from the TSS were identified, and the gene promoters regions were parsed into those that have exclusively H3K4me3 peaks (H3K4me3-exclusive), exclusively H3K27me3 peaks (H3K27Me3-exclusive) and those with overlapping H3K4me3 and H3K27me3 (bivalent). The distances of the center of the peak calls and overlapping regions from the TSS are plotted in E, F and G. The distribution of the distances from the TSS is plotted to depict the probability of occurrence of the peaks at varying distances from the TSS (x-axis, 0 position) for H3K4me3-exclusive promoters (E), H3K27Me3-exclusive promoters (F) and bivalent promoters (G). H, Top panel: Input sequencing reads to show the nucleosome-free zone. Bottom panel shows sequencing reads downstream from the TSS relative to the symmetric position upstream from the TSS as Log2 ratios (y-axis). Depletion in sequencing reads in the 0–1000 bp region for H3K4Me3-exclusive (black) and bivalent promoters (green) is more prominent compared to the H3K27me3-exclusive (red) promoters and promoters marked with none (blue) of the marks analyzed here. (JPG 547 kb) [file 12920_2016_221_MOESM3_ESM.jpg]

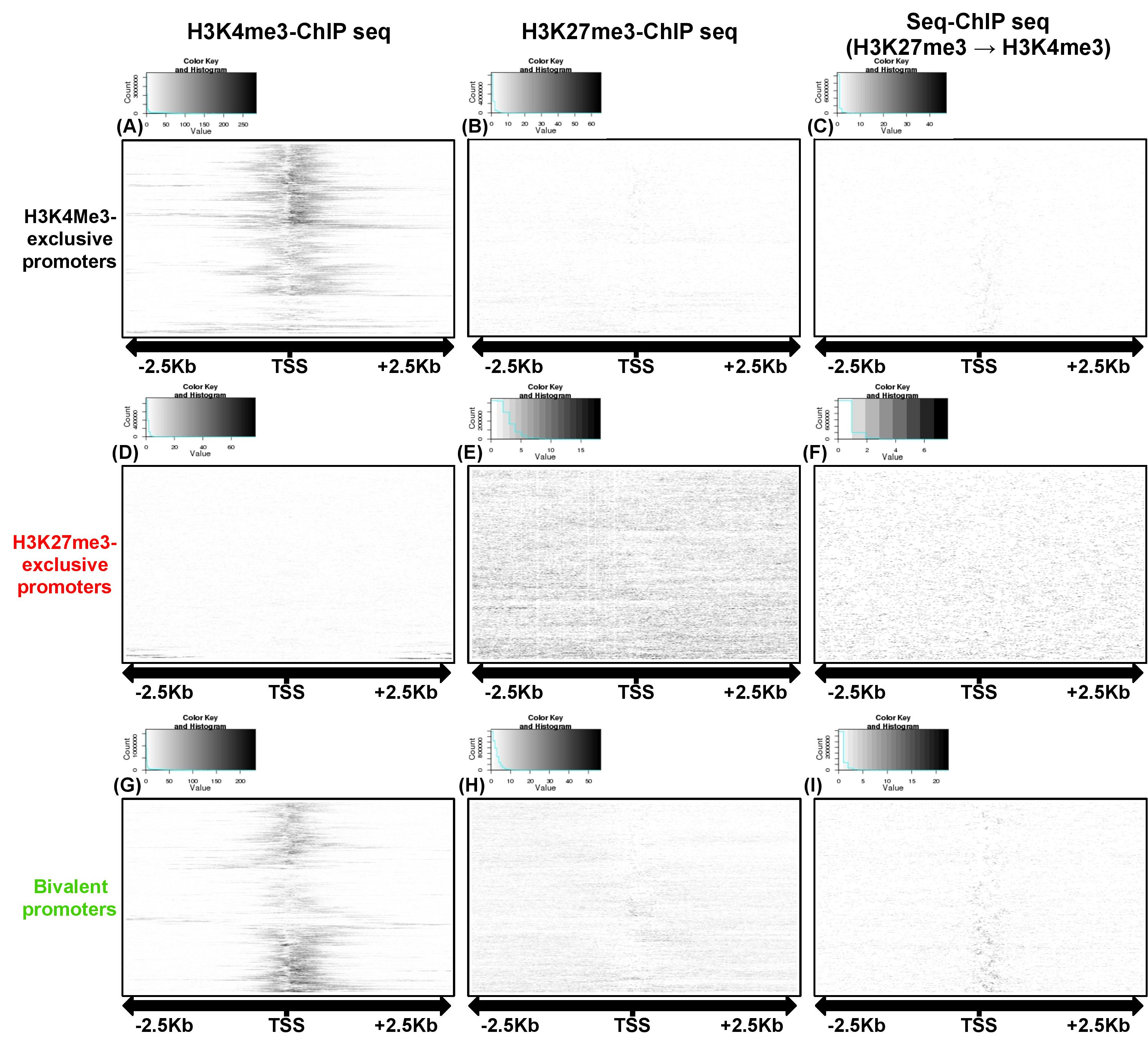

Supplement: Additional file 4: Figure S4. — Heat maps comparison of single ChIP versus sequential ChIP data. Gene level plots as heatmaps showing input normalized ChIP-seq reads in +/− 2.5Kb window around the TSS. Genes in each panel is ordered according to the increasing read counts (top to bottom) of the Sequential ChIP (H3K27me3 → H3K4me3)-seq reads for each category of promoters (from Fig. 2h, labeled to the left of each row). Scale bar shows color scale of normalized read counts. (JPG 717 kb) [file 12920_2016_221_MOESM4_ESM.jpg]

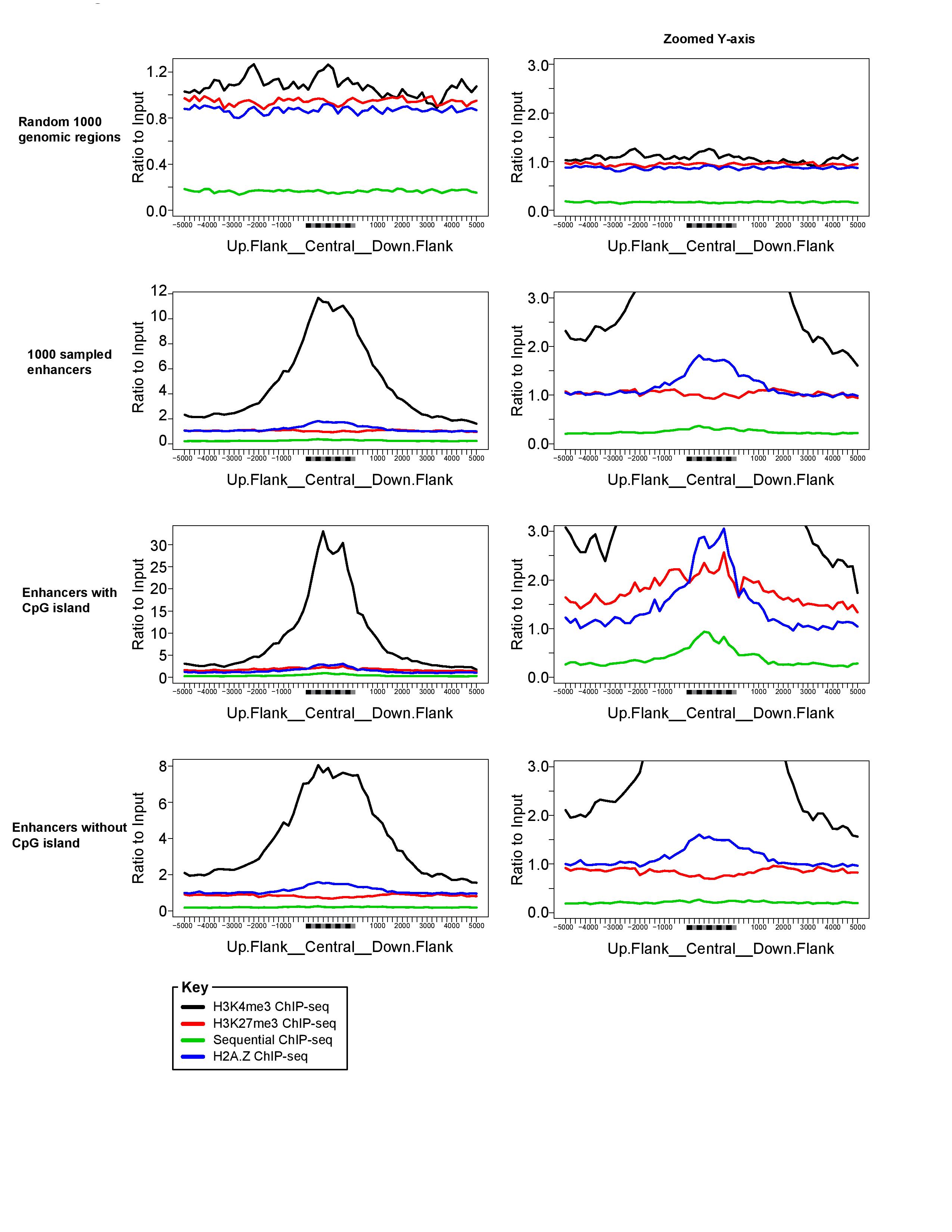

Supplement: Additional file 5: Figure S5. — Analysis of enhancer regions based on CpG islands. Distribution of the H3K4me3, H3K27me3, bivalent and H2A.Z mononucleosomes at enhancers and flanking regions. ChromHMM defined enhancers in human embryonic stem cells (ENCODE project) was downloaded from UCSC and subtracted for Refseq promoter regions. Average profile of the input normalized sequencing coverage data for a random set of 1000 genomic elements (1st row) and 1000 enhancer elements (2nd row), divided into 10 intervals, and their flanking regions (detailed in methods) are plotted. 3rd and 4th row show the profiles for the enhancer elements subsetted, respectively, by presence or absence of CpG-island (s) at the enhancers. Plots on the right column are identical to those on the left except that the y-axis is zoomed to show the typically lower enrichment of H3K27, H2A.Z and bivalent nucleosomes (compared to H3K4me3). (JPG 581 kb) [file 12920_2016_221_MOESM5_ESM.jpg]

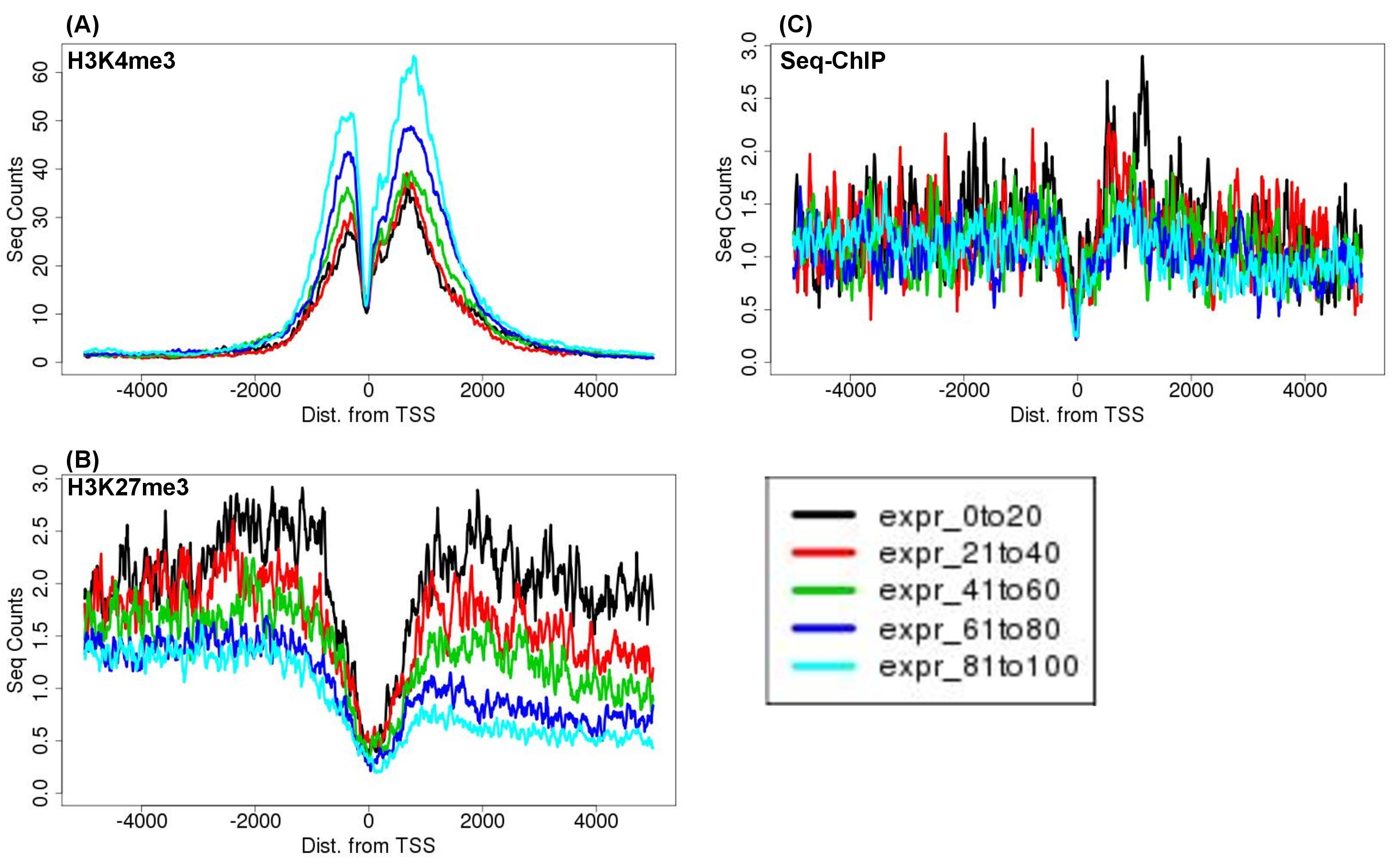

Supplement: Additional file 6: Figure S6. — Distribution of raw ChIP-seq reads for H3K4me3, H3K27me3 and sequential ChIP-seq. Average of raw reads from H3K4me3 ChIP-seq (A), H3K27me3 ChIP-seq (B) and Sequential ChIP (H3K27me3 → H3K4me3)-seq (Seq-ChIP-seq) reads (C) for the groups of genes sub-setted into quintiles (0–20, 21–40, 41–60, 61–80, 81–100) of increasing expression levels (obtained from gene expression microarray data) as shown in color key. The Seq-ChIP-seq reads for the different expression quintiles are not correlated with that of the H3K4me3 enrichment, which shows a decreasing enrichment as the gene expression levels decrease. The lack of correlation between H3K4me3 and Seq-ChIP-seq reads for the different gene expression quintiles indicate that the Seq-ChIP-seq reads are not non-specific flow-through of H3K4me3 in the primary ChIP with anti-H3K27me3. (JPG 380 kb) [file 12920_2016_221_MOESM6_ESM.jpg]
