# Supplementary material for: Genome-wide positioning of bivalent mononucleosomes
Source: BMC Med Genomics. 2016 Sep 15;9:60. doi: 10.1186/s12920-016-0221-6 (PMC5025636; doi:10.1186/s12920-016-0221-6)
Supplement: Additional file 7: — Supplemental Methods. (DOCX 15 kb) [file 12920_2016_221_MOESM7_ESM.docx]

**SUPPLEMENTAL METHODS**

The following primers were used for PCR analysis of DNA obtained by ChIP:

| MLH 1 | SetA-f | agaaaggccgcaaggggagaggag |
| --- | --- | --- |
|  | SetA-r | gctactgcccgctaccta |
|  | SetB-f | cgctgaagggtggggctggatgg |
|  | SetB-r | gtctagatgctcaacggaagtgc |
| CDO1 | SetA-f | tcttggacttattttcacctagcc |
|  | SetA-r | tgcgaaaattgaggatggat |
|  | SetB-f | ctgaacctgatttgtgtgtgc |
|  | SetB-r | gtgccgaaacgtaaggatgt |
| SFRP1 | SetA-f | aagtttgggaggccaagg |
|  | SetA-r | atgcccccgctaattttt |
|  | SetB-f | ggactgcgccttttgtcc |
|  | SetB-r | ctctgcgccctgttctcc |
| SOX17 | SetA-f | gcagttccttggaagtaaactagg |
|  | SetA-r | aacataccgagcgtccattc |
|  | SetB-f | gtgatgggtgagcggaaag |
|  | SetB-r | gagggagatctgggactcct |
| MYC | Forward | ccgcctgcgatgatttatac |
|  | Reverse | gctccctctcaaaccctctc |
